# Physicochemical, Structural, and Digestive Properties of Green Banana Starch from Five Chinese Mutant Banana Species

**DOI:** 10.3390/foods14040706

**Published:** 2025-02-19

**Authors:** Dexian Yuan, Yutong Zhang, Xiaoai Chen, Fei Xu, Kexue Zhu, Jinling Wang, Yanjun Zhang

**Affiliations:** 1 College of Life Sciences, Northeast Forestry University, Harbin 150040, China; 2 Spice and Beverage Research Institute, Chinese Academy of Tropical Agricultural Sciences, Wanning 571533, China; 3 National Center of Important Tropical Crops Engineering and Technology Research, Wanning 571533, China; 4 Key Laboratory of Processing Suitability and Quality Control of the Special Tropical Crops of Hainan Province, Wanning 571533, China

**Keywords:** banana starch, physicochemical properties, particle morphology properties, digestive properties, functional properties

## Abstract

This study provides a comprehensive analysis of the physicochemical, structural, and functional properties of starches extracted from five distinct banana varieties. The starches were labeled as follows: ‘Nan Tian Huang’ starch (NS), ‘Da jiao’ starch (DS), ‘Gui jiao’ starch (GS), ‘Gong jiao’ starch (OS), and ‘Hong jiao’ starch (HS). The results show that all starches have A-type crystalline structures and contain high levels of resistant starch, ranging from 88.3% to 93.5%. The amylose content ranges from 21.97% to 55.46%. The starches isolated from the five banana varieties are predominantly flat, rod-shaped, and spherical. Particle sizes vary significantly, ranging from 19.75 to 28.65 µm, which contributes to differences in their functional properties. For example, DS demonstrates exceptional functional properties, including high RS content, a low glycemic index, and excellent thermal stability. In contrast, HS starch, despite its high amylose content, exhibits higher enzymatic digestibility and lower freeze–thaw stability. Principal component analysis and correlation analysis revealed that amylose content, thermal properties, and particle morphology are key determinants of the physicochemical and digestive properties of banana starch, emphasizing their interdependence. Additionally, notable differences were observed in the gelatinization properties, thermal characteristics, crystallization, and textural parameters. These findings offer valuable insights into the potential applications of banana starch in functional foods and industrial products, highlighting the importance of starch type in optimizing its functionality.

## 1. Introduction

Banana (*Musa paradisiaca*), a member of the family *Musaceae* and genus *Musa*, is a tropical plant cultivated extensively for its edible fruit. Bananas are rich in nutrients, aromatic in flavor, and widely favored worldwide. Their low investment requirements, high efficiency, and rapid economic returns have established bananas as one of the most important global crops. Banana production serves as a significant source of employment in many developing nations [1]. However, as climacteric fruits, bananas are highly perishable and difficult to store for extended periods. While banana cultivation and consumption have expanded rapidly, the industry faces challenges, including substantial postharvest losses, limited processing technology, and inconsistent quality standards [2]. These issues often result in surplus bananas rotting, leading to significant resource waste. Given their high starch content, converting unripe green bananas into starch offers a more stable storage form (lasting 1–2 years) and improves their versatility and utility [3].

Starch is an essential nutrient, primarily sourced from plant fruits, seeds, and tubers. It is a semicrystalline polymer composed of α-glucan molecules, with crystalline regions formed by amylopectin and amorphous regions consisting of amylose and amylopectin branching points [4]. This structure determines key functional properties, including viscosity, gelatinization, and digestibility. These traits are strongly influenced by the botanical source and processing conditions of the starch [5]. Gu et al. [6] conducted an analysis of the physicochemical properties of starch extracted from seeds of 25 tea varieties, Similarly, Shi et al. [7] investigated sweet potato varieties cultivated under different environmental conditions and reported significant differences in their characteristics. Granule morphology, amylose content, and crystallinity are particularly critical in determining its thermal stability, digestibility, and hydration capacity [8]. Starches with higher amylose content typically display greater structural integrity and resistance to enzymatic digestion, making them ideal for applications requiring slow energy release or low glycemic index foods [9]. Banana starch, a nontraditional starch source, has garnered attention due to its high amylose and resistant starch (RS) content. These properties are associated with potential health benefits, including improved glycemic control, enhanced gut microbiota modulation, and reduced risks of chronic conditions, such as type 2 diabetes and obesity, as supported by prior studies [10]. Li et al. [11] studied starch resources with high amylose content from five Chinese mutant banana species, promoting their application in both food and non-food industries. Despite these advances, comprehensive studies addressing the physicochemical and functional properties of banana starch varieties are still scarce.

This study systematically investigates the physicochemical, structural, functional, and digestive properties of starch isolated from five banana varieties. Correlation analysis and principal component analysis (PCA) were conducted to interpret these properties. Additionally, relationships between physicochemical attributes, functional characteristics, and digestion traits were characterized. The findings offer valuable insights into optimizing banana starch applications in functional foods and industrial products, emphasizing their role in addressing postharvest losses and enhancing value-added processing.

## 2. Materials and Methods

### 2.1. Materials

Unripe bananas of five varieties were procured from the Haikou Experimental Station, Chinese Academy of Tropical Agricultural Sciences, ensuring consistent environmental and growth conditions. These varieties included Nan Tian Huang (*Musa AAA Cavendish*), Da Jiao (*Musa paradisiaca* L.), Gui Jiao (*Musa acuminata Colla*), Gong Jiao (*Musa AA*), and Hong Jiao (*Musa coccinea Andr.*). The D-glucose standards were purchased from Solarbio Science & Technology Co., Ltd. (Beijing, China). Other analytical-grade chemicals were procured from Aladdin Biochemical Technology Co., Ltd. (Shanghai, China).

### 2.2. Preparation of Banana Starch

Banana starch was extracted using a water–alkaline method, adapted from Yang et al. [2]. The unripe bananas were peeled and sliced thinly, then soaked in 0.3% (*w/v*) citric acid solution for 20 min to inhibit browning. After immersion, the slices were flash-frozen with liquid nitrogen and stored at −80 °C. The frozen samples were vacuum-dried at 50 °C for 48 h, ground 2–3 times with a high-speed grinder, and sieved through a 100-mesh screen. To extract starch, 100 g of banana flour was mixed with 1 L of distilled water, filtered through a 100-mesh cloth, and centrifuged at 4000× *g* at 15 °C for 10 min. The resulting precipitate was resuspended in 1 L of 0.2% (*w/v*) NaOH solution, stirred for 10 min to remove soluble fibers, and centrifuged again under the same conditions. Exogenous impurities were removed manually, and the starch sediment was dried at 45 °C for 24 h, ground, and sieved through a 100-mesh screen. The starches from Nan Tian Huang, Da Jiao, Gui Jiao, Gong Jiao, and Hong Jiao were labeled NS, DS, GS, OS, and HS, respectively, and stored in sealed containers at room temperature for further analysis.

### 2.3. Proximate Composition of Isolated Starch

The contents of moisture, total starch, ash, lipids, and proteins were determined following the *AOAC Official Methods of Analysis* (18th edition, 2012) [12].

### 2.4. Amylose Content

The amylose content was assessed using a modified method by Li et al. [9]. Dry starch samples (100 mg) were treated with 1 mL of ethanol and 9 mL of 1 M NaOH, heated in boiling water for 10 min, and cooled to room temperature. The solution was diluted to 100 mL with deionized water. A 2.5 mL aliquot was combined with 0.5 mL of 1 M acetic acid and 1 mL of iodine–potassium iodide solution, then diluted with 20 mL of water. The absorbance at 620 nm was measured using a spectrophotometer (SPECORD 250 Plus, Analytik Jena AG, Jena, Germany).

### 2.5. Analysis of Functional Properties

The solubility (S) and swelling power (SP) were determined following the method by Kajubi et al. [13] with minor modifications. A 2% starch suspension was incubated at 55, 65, 75, 85, and 95 °C for 30 min, cooled to room temperature, and centrifuged at 3800 rpm for 15 min. Soluble starch in the supernatant was dried at 105 °C to calculate solubility, while the sediment was weighed to measure swelling power. The following equations are used:

$$S\left(\%\right)=\frac{A}{W}\times 100$$

$$\mathrm{SP}\left(g/g\right)=\frac{P}{W\left(1-S\right)}\times 100$$

where A is the weight of the dried supernatant, W is the sample weight (dry basis), and P is the weight of the sediment.

The freeze–thaw stability of banana starch was measured following the method of Zhong et al. [14] with slight modifications. The starch suspension (6%) was incubate at 90 °C for 30 min in a water bath, then freezed the gelatinized sample at −25 °C for 24 h, and thawing at 30 °C for 4 h. After one treatment cycle, the frozen and thawed samples were subjected to another freeze–thaw treatment using the same cycle, for a total of five treatment cycles. Subsequently, centrifuged at 4000 rpm for 15 min, weighed the sediment. The syneresis rate is used to express the degree of dewatering and shrinkage.

$$\mathrm{Syneresis}\;\left(\%\right)=\frac{\mathrm{weight}\;\mathrm{of}\;\mathrm{the}\;\mathrm{gelatinized}\;\mathrm{starch}}{\mathrm{weigh}\;\mathrm{of}\;\mathrm{the}\;\mathrm{sediment}}\times 100$$

### 2.6. Morphology and Particle Size Distribution Analysis of Starch Granules

Various types of banana starch were affixed to sample holders using double-sided conductive adhesive. Following vacuum gold sputtering, the samples were analyzed using a scanning electron microscope (SEM) (Phenom ProX, Rotterdam, The Netherlands) at an accelerating voltage of 10 kV. The morphology of starch granules was observed at a magnification of 2000× [15].

The particle size distribution of unripe banana powder was measured using a dynamic light scattering instrument (Mastersizer 3000, Malvern Instruments Ltd., Malvern, UK). Starch samples were prepared by mixing with ultrapure water at a concentration of 0.01% (m/V) under ambient conditions. The refractive indices were set to 1.33 for the dispersant and 1.53 for the sample, with an obscuration parameter maintained at 1–2%. Particle sizes corresponding to cumulative distributions of 10%, 50%, and 90% (Dx(10), Dx(50), and Dx(90)) were recorded, representing the diameters at which these cumulative percentages were reached. The surface area mean diameter (D[3,2]) and volume mean diameter (D[4,3]) were also calculated [16].

### 2.7. X-Ray Diffraction Analysis

Crystallization patterns of starch were analyzed with an X-ray diffractometer (Bede XRD Di System, UK), scanning at 2θ angles from 4° to 40° with 0.02° steps at 10°/min. Crystallinity (RC) was quantified using Jade 6.0 software [11]:

$$\mathrm{RC}\left(\%\right)=\frac{A_{c}}{A_{c}+A_{a}}\times 100$$

where A_c_ is the area of the crystalline region and A_a_ is the area of the amorphous region.

### 2.8. Pasting Properties

A Rapid Visco Analyzer (RVA Super 4, Newport Scientific, Warriewood, Australia) assessed pasting properties. Starch (3 g) was mixed with 25 mL of deionized water, stirred at 960 rpm for 10 s, and analyzed using the Standard 1 program. Heating from 50 °C to 95 °C (6 °C/min), holding at 95 °C for 5 min, cooling back to 50 °C, and holding for 2 min determined viscosity parameters [17].

### 2.9. Thermal Properties

Thermal behavior was examined with a differential scanning calorimeter (DSC-Q2000, TA Instruments, New Castle, DE, USA). Samples (100 mg dry basis) mixed with 10 mg water were incubated for 24 h at room temperature before heating from 10 °C to 100 °C at 10 °C/min [18].

### 2.10. Fourier Transform Infrared (FTIR) Spectrum

Short-range molecular structures were characterized using FTIR spectroscopy (Nicolet 6700, Thermo Fisher Scientific, Branchburg, NJ, USA). Scans with a resolution of 4 cm^−1^ (64 scans per sample) recorded the absorbance ratio between 1047 cm^−1^ and 1022 cm^−1^ [19].

### 2.11. Gel Texture Properties of Starch

The gel texture was analyzed using a texture analyzer (TA. XT Plus, Texture Technologies, Port Talbot, UK). A 10% starch suspension was heated to 100 °C with continuous stirring at 180 rpm for 10 min. The temperature was then maintained at 100 °C for an additional 15 min. The starch paste was cooled at a rate of 10 °C/min to 20 °C and stored at 4 °C for 24 h to allow gelation. The resulting gel was shaped into cylindrical samples with a radius of approximately 1.5 cm and a height of 4 cm. The resulting gel was shaped into cylindrical samples with a radius of approximately 1.5 cm and a height of 4 cm. A P/36r probe was used in TPA mode with a strain force of 50%. The initial, testing, and final speeds were all set to 1.0 mm/s [20].

### 2.12. In Vitro Digestion Analysis

To analyze in vitro digestion were determined following the method by Li et al. [9], 200 mg of starch sample was precisely weighed into a flask, followed by the addition of 15 mL of 0.2 mol/L sodium acetate buffer solution (pH 5.2). The mixture was preheated for 5 min in a water bath set at 37 °C. A 10 mL enzyme mixture—consisting of porcine pancreatic α-amylase (290 U/mL) and amyloglucosidase (15 U/mL), both preheated to 37 °C for 5 min—was then added. Immediately, the flask was placed in a 37 °C water bath and shaken at 150 rpm, and digestion times were recorded precisely. At specific intervals (0, 5, 10, 15, 20, 30, 45, 60, 90, 120, 150, and 180 min), 0.5 mL samples were collected into centrifuge tubes, quickly mixed with 4 mL of anhydrous ethanol, and centrifuged at 4000 rpm for 10 min. Glucose content was measured using the DNS method. The mass percentages of rapidly digestible starch (RDS), slowly digestible starch (SDS), and resistant starch (RS) were calculated as follows:

$$\mathrm{RDS}\left(\%\right)=\left(G_{20}-G_{F}\right)\times 0.9/\mathrm{TS}$$

$$\mathrm{SDS}\left(\%\right)=\left(G_{120}-G_{20}\right)\times 0.9/\mathrm{TS}$$

$$\mathrm{RS}\left(\%\right)=\left[\mathrm{TS}-\left(\mathrm{RDS}+\mathrm{SDS}\right)\right]/\mathrm{TS}$$

where G_F_ is the amount of free glucose in the starch before enzymatic hydrolysis (mg); G_20_ is the glucose content after 20 min of hydrolysis (mg); G_120_ is the glucose content after 120 min of hydrolysis (mg); and TS is the total starch content in the sample (mg).

### 2.13. Enzymatic Kinetics

The enzymatic digestion curve of starch samples follows a first-order kinetic equation [21]:

$$C_{t}=\left(C_{\infty }-C_{0}\right)\times \left(1-e^{-\mathrm{kt}}\right)+C_{0}$$

where C_t_ and C_0_ are the starch digestion rates at times t and 0 min, respectively; C_∞_ is the starch hydrolysis rate at the end of digestion; and k is the starch digestion rate constant.

Different stages of digestion were determined using the log of slope (LOS) analysis method, with the following transformation formula:

$$\ln\frac{{\mathrm{dC}}_{t}}{d_{t}}=\ln\left(C_{\infty }-C_{0}\right)-\mathrm{kt}$$

The values of k and C_∞_ were obtained by nonlinear least-squares (NLLS) fitting.

### 2.14. Postprandial Glycemic Response of Starch

The glycemic index (GI) quantifies the relative ranking of foods based on their postprandial glycemic response compared to a standard reference, typically white bread. The primary factor influencing GI is the rate at which carbohydrates are digested and absorbed. To assess the GI, the ratio of the area under the curve (AUC) of the starch sample to that of white bread was calculated using a first-order reaction equation [21]:

$$\mathrm{AUC}=C_{\infty }\left(t_{f}-t_{0}\right)-\left(C_{\infty }/k\right)\left[1-\exp-k\left(t_{f}-t_{0}\right)\right]$$

where C_∞_ is the equilibrium concentration at 180 min, t_f_ is the final time (180 min), t_0_ is the initial time (0 min), and k is the kinetic constant. The hydrolysis index (HI) was obtained by dividing the AUC of the sample by the AUC of the reference (white bread) [9]:

$$\mathrm{HI}=\frac{\mathrm{AUC}\left(\mathrm{sample}\right)}{\mathrm{AUC}\left(\mathrm{white}\;\mathrm{bread}\right)}$$

The GI was then calculated using the following equation:

$$\mathrm{GI}=39.71\times \left(0.549\times \mathrm{HI}\right)$$

### 2.15. Principal Component and Correlation Analysis

Principal component analysis (PCA) was applied to reduce dataset dimensionality and identify major variables contributing to the observed variation. The analysis was performed using SPSS 12.0.1 (SPSS Inc., Chicago, IL, USA), with data scaled and normalized to ensure consistency across variables. Correlation analysis assessed the strength and direction of linear relationships between variables. Pearson’s correlation coefficient (*r*) was used for normally distributed data, while Spearman’s rank correlation was applied for non-parametric datasets. Statistical significance was defined as *p* < 0.05. Heatmaps were used to visualize correlation matrices, emphasizing key patterns and relationships [11].

### 2.16. Statistical Analysis

Mean values, standard deviations, significant differences, and correlations between parameters were calculated using SPSS 12.0.1 (SPSS Inc., Chicago, IL, USA). Significant differences between means were assessed using Duncan’s multiple range test with a significance level of 0.05. Graphical representation and starch digestion kinetics fitting were conducted using Origin 2022 (OriginLab Corporation, Northampton, MA, USA).

## 3. Results and Discussion

### 3.1. Proximate Composition

The proximate composition of starches extracted from five different varieties of green banana is summarized in Table 1. The moisture content of all samples ranged from 2–3%, well below the recommended commercial starch moisture level of less than 20%, as reported by Nwokocha et al. [22]. These values were lower than those reported for twelve varieties of buckwheat starch (8.43–13.65%) by Kongolo et al. [18] and comparable to the 4.5–9.65% range observed for banana starch by Olawoye et al. [23]. Moisture content is influenced by factors such as drying temperature and the relative humidity during processing and storage [23]. Protein levels in all starches were below 1.0%, aligning with the *Codex Alimentarius* and Chinese food industry standards for high-purity starch [9]. The fat and ash contents ranged between 0.15 and 0.42% and 0.07 and 0.13%, respectively, consistent with values reported by Li et al. [9]. These low levels of moisture, fat, protein, and ash confirm the high purity of the starches analyzed. The DS variety exhibited the highest total starch content (98.59%) and the highest purity among the varieties tested.

The amylose content of starches from the five banana varieties ranged from 21.97% to 55.46%. Significant differences were observed among the varieties, with HS exhibiting the highest amylose content (55.46%) followed by DS (49.94%). In contrast, GS displayed the lowest amylose level (21.97%). No significant difference (*p* > 0.05) was observed between the amylose contents of NS (30.42%) and OS (30.67%). These findings align with trends reported by Paramasivam et al. [24] but indicate higher amylose content than the values reported by Ssonko et al. [25], who found amylose levels in banana cultivars from Uganda to range from 11.96% to 12.59%. Variations in amylose content across studies may result from differences in environmental factors, such as climate, soil composition, and agricultural practices. High-amylose starches are known to provide health benefits akin to dietary fiber and hold promise as functional food ingredients due to their potential to lower the glycemic index [6,26,27].

### 3.2. Morphology and Particle Size Distribution Analysis of Starch Granules

**Figure 1:**
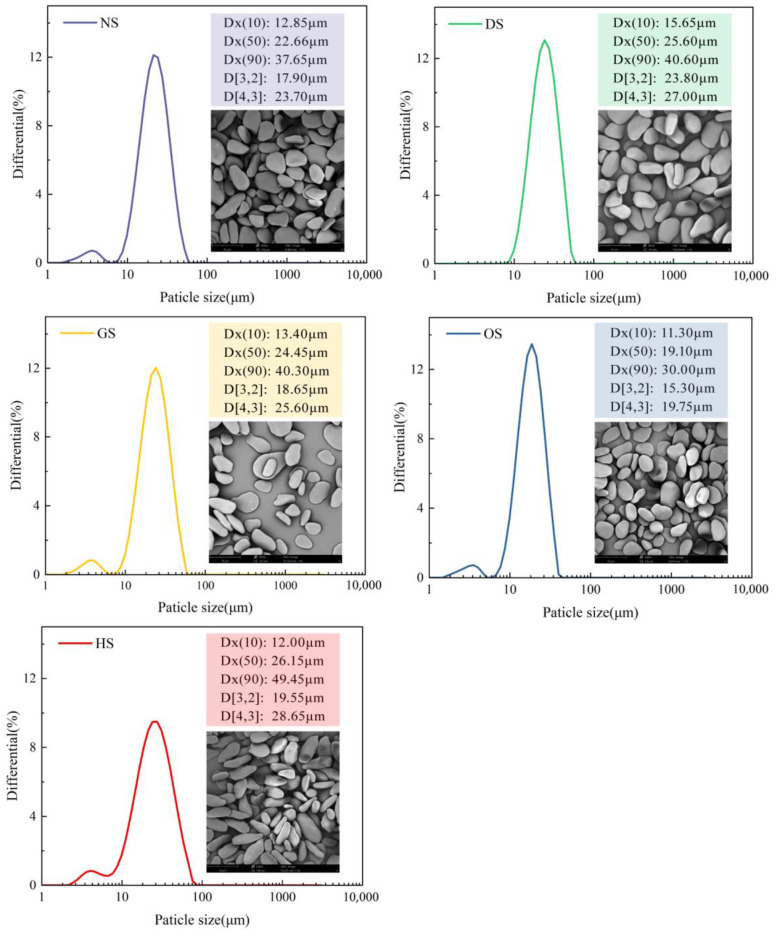
Particle size distribution and microscopy of starch granules at 2000× magnification (SEM).

Starch granules from five banana varieties exhibit distinct morphological characteristics, as illustrated in Figure 1. Granules from NS were predominantly flattened and spherical with smooth surfaces and lacked noticeable depressions, reflecting an irregular morphology. DS granules shared similar flattened and spherical shapes but were slightly larger and more elongated. GS granules were bell-shaped, a distinctive feature among the samples. OS granules were the smallest, with a predominantly flattened and spherical shape and more pronounced irregularities. In contrast, HS granules were primarily rod-shaped, significantly differing from the shapes of the other varieties. These morphological differences likely arise from the genetic and environmental factors unique to each banana cultivar. The variation in granule shape and surface properties influences the functional characteristics of starch, such as gelatinization, swelling, and water absorption capabilities. Our findings diverged from those observed in corn starch samples, which exhibited polygonal shapes characterized by multiple planar and angular surfaces [28]. This variation is likely due to differences in amylose content and the specific nature of the X-ray crystalline structure. In comparison to banana starch granules described by Li et al. [11], the granules analyzed in this study exhibited notably smoother surfaces. The enhanced smoothness might result from smaller, spherical clusters formed by polymerized branched starch nanomodules. These clusters are arranged beneath the granule surface in a semi-wafer-shaped configuration, reducing the root-mean-square roughness of the nanosurface [29,30]. Furthermore, particle size distribution parameters—Dx(10), Dx(50), and Dx(90)—represent the particle dimensions below which 10%, 50%, and 90% of the total starch particles fall, respectively [31]. The Dx(10), Dx(50), and Dx(90) values for all samples ranged as follows: 12.85–37.65 µm, 15.65–40.60 µm, 13.40–40.30 µm, 11.30–30.00 µm, and 12.00–49.45 µm, respectively. HS had the largest granules (Dx(90) = 49.45 µm, D[4,3] = 28.65 µm), while OS displayed the smallest granules (Dx(90) = 30.00 µm, D[4,3] = 19.75 µm). The average particle size (D[4,3]) differed across the starch samples: HS starch exhibited the largest granules (28.65 µm), followed by DS (27.00 µm), GS (25.60 µm), NS (23.70 µm), and OS starch (19.75 µm). The larger granule size of HS likely contributes to enhanced swelling and gelatinization properties, as the increased surface area facilitates greater water absorption and interaction. Differences in size distribution patterns were observed among the starch samples. DS exhibited a unimodal distribution, suggesting uniform granules. In contrast, the other starches displayed bimodal distributions, indicating a heterogeneous mix of small and large granules. The large granules of HS and DS suggest a higher water-binding capacity, which can enhance swelling power and contribute to improved gelatinization and texture. These characteristics make HS and DS suitable for applications requiring high swelling and viscosity, such as in thickening or gelling agents. Smaller granules, such as those of GS, may be preferable for applications requiring higher stability or lower retrogradation rates. Smaller granules typically have a more compact crystalline structure, making them better suited for low-gelatinization products or formulations that demand enhanced freeze–thaw stability.

### 3.3. Crystalline Structure

**Figure 2:**
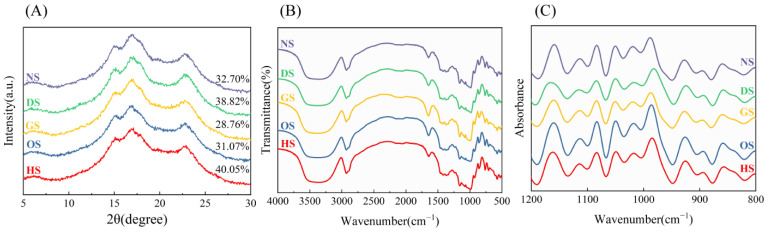
Ordered structure of banana starch: (**A**) X-ray diffraction patterns of starch samples; (**B**) Fourier transform infrared spectra; (**C**) Deconvoluted infrared spectra.

The long-range order and crystalline arrangements of starch granules were examined through X-ray diffraction (XRD), as depicted in Figure 2A. The diffraction patterns revealed distinctive peaks near 15°, 17°, 18°, and 23°, characteristic of A-type crystalline structures typically observed in cereal starches [32]. The degree of crystallinity for starches derived from the five banana varieties was quantified, ranging from 28.76% to 40.05%. HS exhibits the highest crystallinity (40.05%), reflecting a well-ordered crystalline region. This structural feature likely enhances its functional properties, such as increased resistance to enzymatic hydrolysis [33]. Conversely, GS demonstrates the lowest crystallinity (28.76%), indicating a predominantly amorphous structure, which may improve its gelatinization and solubility properties [32]. Variations in crystallinity among the starches can be attributed to genetic differences between banana cultivars, as well as differences in their amylose and amylopectin compositions. Higher crystallinity is typically associated with a greater proportion of amylopectin, which forms double helical structures that align into ordered crystalline regions [7]. However, the higher proportion of amylose in HS may promote its crystallinity by forming an ordered structure under certain conditions (such as during cooling or regeneration). Additionally, the higher amylose content may enhance the degree of branching within amylopectin, potentially leading to the formation of more helical structures, which in turn could result in a higher degree of crystallinity [34].

### 3.4. Short-Range Ordered Structure

The FTIR spectra identify characteristic absorption bands corresponding to functional groups in the starch molecules. The absorbance ratios of 1045/1022 cm^−1^ (DO) and 1022/995 cm^−1^ (DD) reflect the ratio of crystalline to amorphous regions and the density of double-helical structures, respectively [35]. As depicted in Table 2, DS again shows the highest DD value (4.67 ± 0.03), suggesting a high density of double-helical structures. This structural characteristic enhances the stability and resistance to enzymatic hydrolysis of DS, making it particularly suitable for applications requiring slow digestion or resistance to retrogradation. HS starch also has a high DD value (4.36 ± 0.06), which aligns with its high crystallinity and well-ordered molecular structure. This finding suggests strong hydrogen bonding and tightly packed amylopectin chains. The lower double-helix content in OS starch indicates a more disordered molecular structure, which could improve its solubility and swelling power during gelatinization [36].

### 3.5. Solubility, Swelling Power and Freeze–Thaw Stability

**Figure 3:**
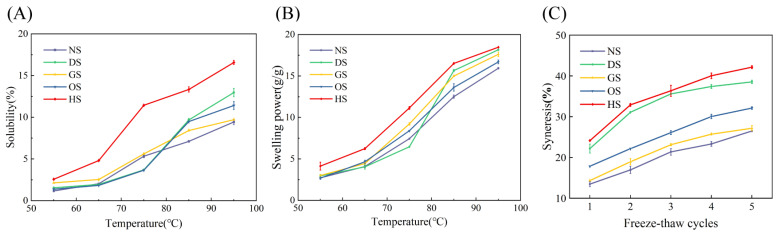
Solubility (**A**), swelling power (**B**), and freeze–thaw stability (**C**) of starch isolated from five different varieties of banana.

The solubility of the five banana starch varieties (NS, DS, GS, OS, and HS) increases with temperature, as illustrated in Figure 3, which is consistent with most reports [37]. This trend is attributed to the gradual disruption of intermolecular and intramolecular hydrogen bonds within the starch granules, which leads to the breakdown of the crystalline structure. This process exposes more hydroxyl groups in starch molecules, facilitating their interaction with water and enhancing solubility [38]. The low solubility observed at 55 °C and 65 °C is due to the intact crystalline regions within the starch granules, which preserve their molecular integrity and limit water penetration and solubility [39]. HS exhibits significantly higher solubility compared to the other varieties (*p* < 0.05). This higher solubility is likely due to the larger granule size and higher amylose content of HS starch, which facilitate granule disruption and enhance solubilization at elevated temperatures [40]. In addition, although DS starch also has a higher amylose content and large granule size, its solubility remains low between 55 °C and 75 °C but increases sharply near its gelatinization temperature. This can be attributed to the dense and compact structure of DS starch granules and incompletely gelatinized at low temperatures, limiting water penetration and molecular chain dissolution. As the temperature rises, the granule structure gradually breaks down, releasing more amylose and significantly increasing solubility [37].

Similar to previous reports [15], the swelling power of the five banana starch varieties also increases with rising temperature. Swelling occurs when starch granules absorb water, leading to the separation of amylose and amylopectin. This process is facilitated by the breakdown of hydrogen bonds at higher temperatures [41]. At 55 °C and 65 °C, the swelling power remains low, indicating the presence of strong intermolecular forces that resist water penetration. As the temperature increases, the crystalline regions disintegrate, enabling greater water binding and granule expansion [42]. HS demonstrates the highest swelling power (*p* < 0.05), likely because its larger granules offer a greater surface area for water interaction. The presence of short-chain amylopectin may also positively influence swelling power, while the high amylose content further enhances water binding [43]. Notably, DS exhibits a sharp increase in swelling power near its gelatinization temperature. This behavior suggests that DS granules reach a critical point at their gelatinization temperature, where rapid disintegration occurs, exposing amylose and amylopectin to water.

The syneresis rates during repeated freeze–thaw cycles reflect the stability of the starch gels. Syneresis rates increase with the number of freeze–thaw cycles, indicating a progressive weakening of starch–water interactions [44]. All starch varieties exhibit an increase in syneresis rates as freeze–thaw cycles progress. This increase is attributed to retrogradation, a process in which starch molecules realign and expel water from the gel matrix [45]. HS exhibits the highest syneresis, indicating the lowest freeze–thaw stability. The high amylose content and larger granule size of HS starch likely contribute to its rapid retrogradation and water separation. DS exhibits slightly better freeze–thaw stability compared to HS, NS, GS, and OS, which display relatively lower syneresis rates and better freeze–thaw stability, likely due to their smaller granule sizes and moderate amylose content, which reduce the extent of retrogradation.

### 3.6. Thermal Properties

**Figure 4:**
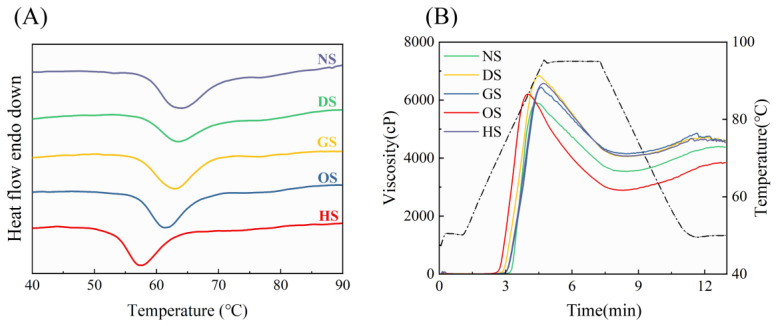
Thermodynamic characteristics (**A**) and pasting profiles (**B**) of starch isolated from five different varieties of banana. Note: The dashed black line in subfigure (**B**) represents the temperature curve.

Thermal properties are essential for understanding the gelatinization behavior of starch, which is a key factor in its application across food systems. Differential scanning calorimetry (DSC) was used to analyze starches from five banana varieties, with the results presented in Figure 4 and Table 3. The To (onset temperature), Tp (peak temperature), Tc (conclusion temperature), and ΔH (enthalpy) of these starches ranged from 57.11 to 60.22 °C, 57.33 to 65.53 °C, 62.82 to 76.32 °C, and −12.40 to −5.95 J/g, respectively, aligning with findings reported by Yang et al. [2]. The thermal properties of starches are influenced by factors such as growth conditions, variety, and maturity stage. Among the varieties, NS starch exhibited the broadest gelatinization temperature range (16.10 ± 0.98 °C), indicating greater heterogeneity in its starch granules. This broad range may reflect structural variations in the arrangement of starch components within the granules. These observations are consistent with previous findings from scanning electron microscopy. HS demonstrates lower transition temperatures (To, Tp, and Tc). According to Zhang et al. [46], this may be attributed to the larger particle size of HS starch, which leads to the formation of more branched starch crystals. However, these crystals are less ordered, resulting in a less perfect structure and reduced morphological stability. Consequently, the thermal stability of these starch granules is more uneven compared to those with smaller particle sizes.

### 3.7. Pasting Properties

Pasting properties describe the behavior of starch during heating and cooling, which profoundly influences the quality and applications of starch-based foods. Key determinants include particle size, porosity, amylose content, and molecular structure [35,47]. Significant differences in pasting viscosity parameters among the starches were observed using Rapid Visco Analyzer (RVA) profiles, as shown in Figure 4B and Table 3.

NS and GS exhibited higher peak viscosities (6803.50 and 6567.50 cP, respectively), reflecting superior water-binding capacity and swelling behavior during heating. In contrast, DS had a lower peak viscosity (5895.00 cP), consistent with its weaker granular structure, as previously reported by Alimi et al. [48]. Breakdown viscosity, an indicator of granule resistance to shear stress, reflects thermal stability, with lower values indicating higher stability [49]. Among the starch varieties, breakdown viscosity ranged from 2295.00 to 3310.00 cP. HS exhibited the highest value (3310.00 cP), while OS had the lowest (2295.00 cP), indicating that OS starch is more resistant to heating and shear stresses during cooking.

Setback viscosity reflects the retrogradation tendency and gel-forming ability of starch pastes, with higher values indicating stronger gel formation and increased retrogradation. The setback viscosities of the five banana starch varieties ranged from 459.00 to 955.00 cP. OS exhibited the lowest setback viscosity (459.00 cP), while HS and DS exhibited the highest values (955.00 cP and 840.00 cP, respectively), indicating stronger gel-forming abilities but higher susceptibility to aging.

The pasting temperatures of banana starches ranged from 69.40 °C to 76.70 °C. DS exhibited the highest pasting temperature, likely due to its elevated amylose content and thicker semi-crystalline layer [50] Interestingly, despite HS having a higher amylose content than plantain starch, its pasting temperature was the lowest among the varieties, potentially due to its significantly larger particle size (*p* < 0.05). The lower pasting temperature of HS may be attributed to its significantly larger particle size (*p* < 0.05), as demonstrated by Falade et al. [51], who reported that larger starch particles tend to paste more readily, resulting in reduced pasting temperatures.

### 3.8. Textural Properties

Textural properties are crucial in food and non-food starch applications, significantly influencing sensory and physical qualities of starch-based products. Hardness reflects overall texture quality, while chewiness, resilience, and springiness are indicative of mouthfeel and smoothness [52]. The textural properties of the five banana starch varieties, detailed in Table 3, show that DS and HS exhibited significantly higher hardness, cohesiveness, and gumminess (*p* < 0.05) compared to other varieties. Gel hardness is largely influenced by the crystallization rate of the amylose double helix [53]. Thus, the higher hardness of DS and HS gels may be due to the faster retrogradation of their amylose chains.

### 3.9. In Vitro Digestibility

**Figure 5:**
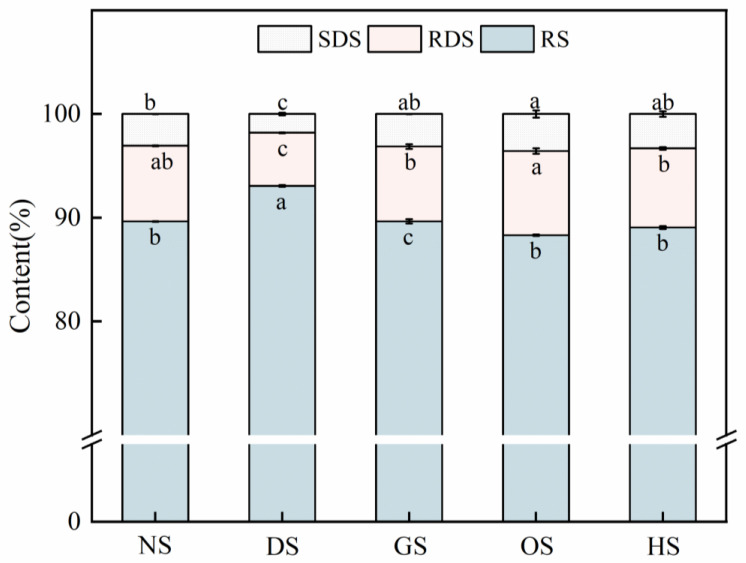
Proportions of RDS, SDS, and RS of starch isolated from five different varieties of banana. Note: Different letters indicate significant differences between data (*p* < 0.05).

Figure 5 illustrates the distribution of RDS, SDS, and RS content among five banana starch varieties. RS content was the predominant fraction, ranging from 53.44% to 62.92%, with DS exhibiting the highest RS level. The low levels of RDS (1.79–3.45%) and SDS (5.06–8.24%) confirm that green banana starches are predominantly composed of RS2-type resistant starch. Significant differences (*p* < 0.05) between varieties are likely attributed to intrinsic factors such as amylose content and granule morphology. DS, with the highest RS content and the lowest RDS and SDS levels, demonstrates structural stability, likely due to its higher crystallinity and compact granular structure [54].

**Figure 6:**
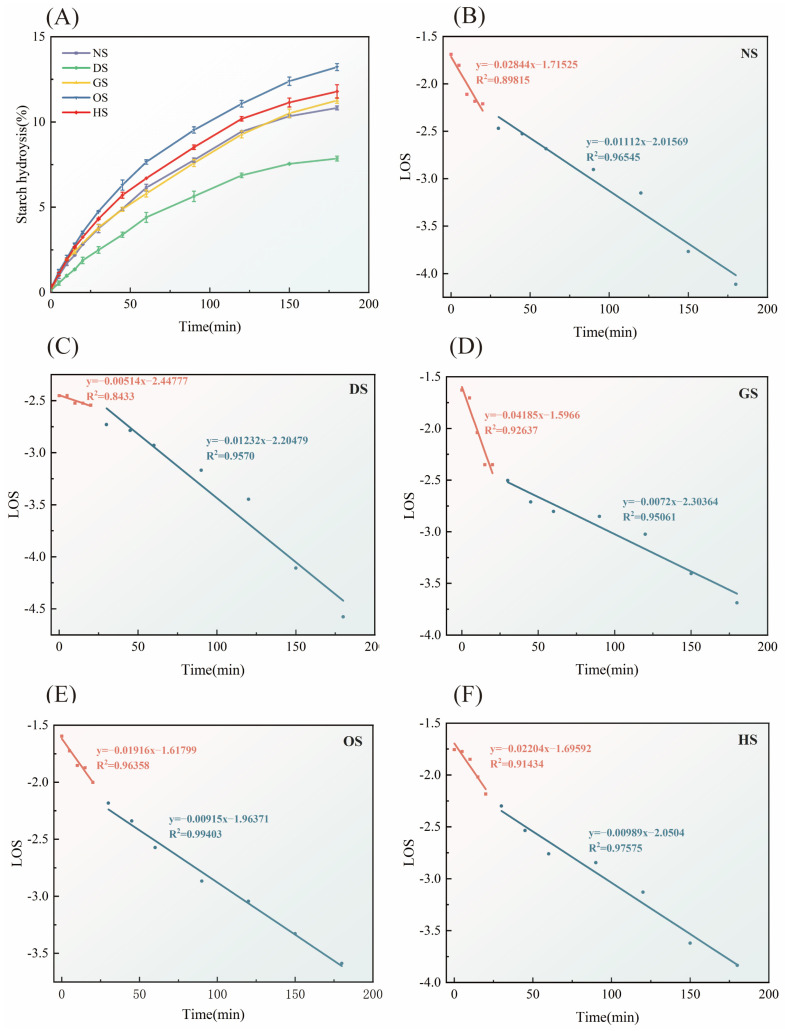
Hydrolysis rates (**A**) and logarithm of slope plot (**B**–**F**) of starch isolated from five different varieties of banana.

The hydrolysis rates and logarithmic slopes of starch isolated from five different banana varieties are presented in Supplementary Table S1. After fitting the data, Figure 6A and Table 4 were generated, illustrating the change in starch hydrolysis rates over time and indicating a gradual release of glucose during the enzymatic digestion process. The hydrolysis process follows first-order kinetics, with a rapid initial increase in hydrolysis within the first 20 min, followed by a plateau phase. The digestion rate constants for rapid (k_1_) and slow digestion (k_2_), along with the final digestibility rate (C_∞_), vary significantly among varieties (*p* < 0.05). DS has the lowest C_∞_, indicating greater resistance to enzymatic digestion. Variability in C_∞_ is linked to structural differences, with higher amylose content and stable crystalline regions reducing enzymatic susceptibility [48,49]. Notably, HS, despite having the highest amylose content, does not show a proportionally lower hydrolysis rate, suggesting that other structural factors may modulate enzymatic resistance.

Figure 6B–F illustrates LOS plots of NS, DS, GS, OS, and HS, respectively, revealing two digestion phases: rapid and slow digestion. Differences in k_1_ and k_2_ constants (*p* < 0.05) among varieties highlight variability in the rates of these phases. DS and NS exhibit the steepest decline during the rapid digestion phase, while GS and OS demonstrate more moderate digestion profiles. LOS analysis provides valuable insights into digestion dynamics, emphasizing the unique enzymatic breakdown behavior of banana starches. The biphasic LOS plots align with earlier findings on plant-based starches, such as buckwheat, where digestion is compartmentalized due to heterogeneous granule structures [8].

The GI values in Table 4 highlight the potential health implications of banana starches. DS, with its low GI value, is well-suited for use in low glycemic index foods, contributing to blood glucose regulation and the prevention of metabolic diseases [55]. Previous studies suggest a negative correlation between amylose content and GI. However, HS, despite its higher amylose content, exhibited a higher GI value, likely due to factors such as particle structure or crystallization properties [56]. Furthermore, NS showed a relatively low eGI value, which may be linked to its uniform particle morphology and moderate RS content.

### 3.10. Principal Component and Correlation Analysis

**Figure 7:**
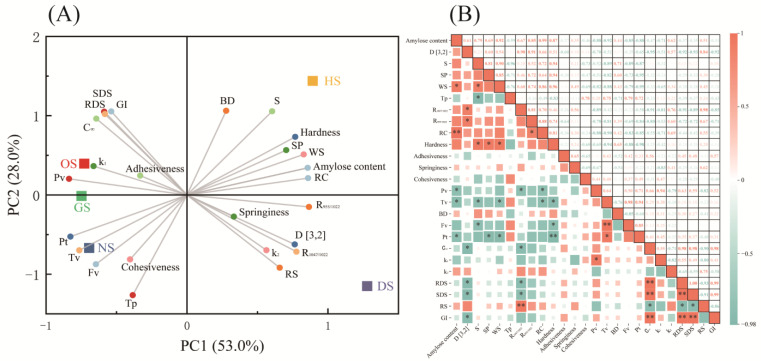
Statistical analysis: Principal component analysis (PCA) score and loading plot of PC1 and PC2 of starch samples. (**A**) Correlation analysis (**B**) of starch isolated from five different varieties of banana. * *p* < 0.05, ** *p* < 0.01, indicating statistically significant differences.

The *PCA* biplot (Figure 7A) effectively distinguishes banana starch varieties based on their physicochemical, functional, and digestibility properties. Principal Component 1 (PC1, 53.0%) and Principal Component 2 (PC2, 28.0%) together explain 81.0% of the total variance, confirming the effectiveness of dimensionality reduction. Each variety forms a distinct cluster, reflecting its unique property profile. For instance, DS is located in the negative quadrants of both PC1 and PC2 due to its high RS content and cohesiveness, making it particularly suitable for low glycemic index applications. In contrast, OS and HS cluster in the positive quadrant of PC1, influenced by their high adhesiveness, SP, and amylose content. These characteristics support their use in industrial applications requiring enhanced viscosity or gel formation. The trait loadings reveal that amylose content, adhesiveness, and SP strongly influence PC1, while digestibility-related traits, such as RS and SDS, primarily define PC2. This differentiation underscores functional trade-offs among varieties, such as between digestibility and mechanical stability, providing guidance for their specific applications.

The correlation heatmap (Figure 7B) reveals statistically significant relationships among key starch properties. Amylose content shows a strong positive correlation with RS (r > 0.8) and hardness, indicating that high-amylose starches form more crystalline regions, enhancing RS while reducing enzymatic digestibility [57]. This characteristic makes DS, with its high amylose content, ideal for functional foods targeting sustained energy release or reduced postprandial glycemic responses. Conversely, amylose content negatively correlates with SP, as increased amylose inhibits excessive water absorption and granule swelling [58].

Swelling power correlates positively with adhesiveness (r ≈ 0.7) and cohesiveness (r ≈ 0.6), highlighting the role of granule hydration in gel integrity and texture. HS and OS, which demonstrate higher SP and adhesiveness, are suitable for applications requiring strong gel formation, such as bakery fillings or industrial thickeners. RDS and SDS exhibit negative correlations with cohesiveness and adhesiveness (r < −0.7 and r < −0.5, respectively), suggesting that enhanced digestibility often compromises mechanical stability. In contrast, *RS* positively correlates with cohesiveness (r > 0.6), reflecting the structural integrity required to resist enzymatic hydrolysis.

These correlations suggest that DS, with its high RS and cohesiveness, is well-suited for low glycemic index products, whereas OS and HS, with higher RDS, are better for quick-energy-release foods. Pasting properties, such as PV and FV, negatively correlate with RDS and SDS but positively with RS. Starches with higher RS, like DS, exhibit superior paste stability and retrogradation, making them appropriate for products requiring extended storage or thermal stability.

## 4. Conclusions

This study systematically evaluated the physicochemical, structural, and functional properties of starches from five banana cultivars, revealing significant differences in their characteristics. For instance, DS demonstrated superior functional properties, such as the highest resistant starch content, the lowest glycemic index, and excellent thermal stability. These characteristics make DS highly suitable for the development of low GI and functional foods. NS has a stable crystal structure and viscosity, with a flexible structure that makes it ideal for products requiring thermal stability and softer gels. GS exhibits excellent thickening properties, making it suitable for thick and stable formulations. PCA and correlation analyses revealed the complex interplay among granule morphology, amylose content, and crystallinity in determining starch functionality. The observed differences in starch properties among the five banana cultivars were attributed to variations in plant origin and structural composition. These findings offer valuable insights for optimizing the application of banana starch in functional foods and industrial products, such as its use as a thickener, gel-forming agent, or component in anti-retrogradation formulations.

## Figures and Tables

**Table 1:** Chemical composition of starch isolated from five different varieties of banana.

Sample	Moisture/%	Protein/(%, db)	Fat/(%, db)	Ash/(%, db)	Total Starch/(%, db)	Amylose Content/(%, db)
NS	2.85 ± 0.01 ^a^	0.30 ± 0.03 ^a^	0.15 ± 0.01 ^c^	0.13 ± 0.00 ^a^	95.11 ± 0.18 ^bc^	30.42 ± 0.77 ^c^
DS	2.20 ± 0.00 ^d^	0.18 ± 0.00 ^b^	0.16 ± 0.00 ^c^	0.07 ± 0.01 ^b^	98.59 ± 0.92 ^a^	49.94 ± 0.15 ^b^
GS	2.38 ± 0.02 ^c^	0.16 ± 0.04 ^b^	0.25 ± 0.06 ^b^	0.08 ± 0.02 ^b^	94.16 ± 0.96 ^c^	21.97 ± 0.31 ^d^
OS	2.25 ± 0.02 ^d^	0.21 ± 0.04 ^ab^	0.16 ± 0.01 ^c^	0.12 ± 0.00 ^a^	96.30 ± 0.14 ^b^	30.67 ± 0.09 ^c^
HS	2.47 ± 0.00 ^b^	0.21 ± 0.10 ^ab^	0.42 ± 0.03 ^a^	0.08 ± 0.02 ^b^	95.41 ± 0.50 ^bc^	55.46 ± 0.60 ^a^

Note: All data were expressed by mean ± SD (*n* = 2). Values with the different letters in the same column are significantly different (*p* < 0.05).

**Table 2:** The short-ordered structures of starch isolated from five different varieties of banana.

Sample	Short-Ordered Parameters	
	DO(R_1047/1022_)	DD(R_955/1022_)
NS	2.55 ± 0.20 ^b^	3.37 ± 0.49 ^b^
DS	3.37 ± 0.04 ^a^	4.67 ± 0.03 ^a^
GS	2.46 ± 0.13 ^b^	3.27 ± 0.13 ^b^
OS	2.49 ± 0.24 ^b^	2.65 ± 0.42 ^b^
HS	2.69 ± 0.10 ^b^	4.36 ± 0.06 ^a^

Note: All data were expressed by mean ± SD (*n* = 2). Values with the different letters in the same column are significantly different (*p* < 0.05). The molecular order (DO) was ascertained by the peak area ratio of R1047/1022 cm^−1^, and the double helicity (DD) was expressed by the peak area ratio of R995/1022 cm^−1^.

**Table 3:** Thermal, pasting, and texture properties of starch isolated from five different varieties of banana.

Properties	Sample				
	NS	DS	GS	OS	HS
To (°C)	60.22 ± 1.02 ^a^	58.26 ± 0.57 ^b^	57.41 ± 0.10 ^bc^	57.11 ± 0.16 ^c^	53.31 ± 0.33 ^d^
Tp (°C)	65.53 ± 0.52 ^a^	63.37 ± 0.12 ^b^	63.03 ± 0.20 ^b^	61.34 ± 0.13 ^c^	57.33 ± 0.11 ^d^
Tc (°C)	76.32 ± 0.13 ^a^	69.85 ± 0.42 ^b^	68.50 ± 0.08 ^c^	66.85 ± 0.09 ^d^	62.82 ± 0.19 ^e^
To-Tc (°C)	16.10 ± 0.98 ^a^	11.59 ± 0.91 ^b^	11.09 ± 0.09 ^b^	9.73 ± 0.09 ^c^	9.51 ± 0.17 ^c^
ΔH (J·g^−1^)	−12.40 ± 0.14 ^d^	−5.95 ± 0.07 ^a^	−7.83 ± 0.08 ^c^	−7.59 ± 0.16 ^b^	−7.48 ± 0.15 ^b^
Peak viscosity (cP)	6568 ± 18 ^b^	5895 ± 98 ^d^	6804 ± 50 ^a^	6448 ± 135 ^b^	6204 ± 43 ^c^
Trough viscosity (cP)	4067 ± 1 ^b^	3540 ± 16 ^c^	4067 ± 16 ^b^	4149 ± 22 ^a^	2893 ± 34 ^d^
Breakdown (cP)	2538 ± 35 ^bc^	2355 ± 86 ^cd^	2720 ± 90 ^b^	2295 ± 140 ^d^	3300 ± 100 ^a^
Final viscosity (cP)	4521 ± 11 ^b^	4380 ± 27 ^c^	4577 ± 1 ^a^	4607 ± 30 ^a^	3848 ± 27 ^d^
Setback viscosity (cP)	464 ± 1 ^c^	840 ± 33 ^b^	510 ± 14 ^c^	459 ± 31 ^c^	955 ± 44 ^a^
Peak time (min)	4.73 ± 0.01 ^a^	4.17 ± 0.03 ^c^	4.50 ± 0.04 ^b^	4.60 ± 0.04 ^ab^	4.00 ± 0.16 ^c^
Setback viscosity (°C)	74.30 ± 0.07 ^b^	76.70 ± 0.85 ^a^	73.10 ± 0.57 ^b^	74.15 ± 0.85 ^b^	69.40 ± 0.71 ^c^
Hardness	148 ± 2 ^d^	245 ± 7 ^b^	193 ± 9 ^c^	188 ± 8 ^c^	299 ± 13 ^a^
Adhesiveness (g·s^−1^)	−467 ± 36 ^b^	−429 ± 42 ^b^	−438 ± 42 ^b^	−166 ± 23 ^a^	−458 ± 26 ^b^
Springiness	0.84 ± 0.00 ^c^	0.98 ± 0.02 ^ab^	0.84 ± 0.04 ^c^	0.98 ± 0.02 ^a^	0.88 ± 0.07 ^bc^
Cohesiveness (g)	0.47 ± 0.01 ^a^	0.43 ± 0.01 ^ab^	0.46 ± 0.02 ^a^	0.40 ± 0.03 ^b^	0.41 ± 0.02 ^b^
Gumminess (g)	70.01 ± 0.31 ^a^	106.94 ± 0.59 ^b^	87.48 ± 0.32 ^c^	75.80 ± 0.23 ^d^	121.73 ± 0.22 ^a^
Cohesiveness	59.04 ± 0.20 ^c^	105.80 ± 1.65 ^a^	73.50 ± 3.38 ^b^	74.64 ± 0.31 ^b^	107.93 ± 0.64 ^a^
Resilience (g)	0.07 ± 0.00 ^a^	0.08 ± 0.00 ^a^	0.06 ± 0.01 ^a^	0.06 ± 0.01 ^a^	0.07 ± 0.03 ^a^

Note: All data were expressed by mean ± SD (*n* = 2). Values with the different letters in the same column are significantly different (*p* < 0.05).

**Table 4:** Kinetic equation characteristics of enzymatic hydrolysis of starches isolated from five different varieties of banana.

Sample	C_∞_ (%)	k_1_ × 10^−2^ (min^−1^)	k_2_ × 10^−2^ (min^−1^)	RDS (%)	SDS (%)	RS (%)	GI
NS	12.25 ± 0.07 ^b^	2.84 ± 0.55 ^b^	1.11 ± 0.09 ^b^	3.06 ± 0.00 ^b^	7.31 ± 0.04 ^ab^	89.63 ± 0.04 ^b^	58.81 ± 0.04 ^c^
DS	9.44 ± 0.30 ^c^	0.51 ± 0.13 ^e^	1.23 ± 0.12 ^a^	1.81 ± 0.31 ^c^	5.13 ± 0.05 ^c^	93.05 ± 0.08 ^a^	53.44 ± 0.39 ^d^
GS	12.86 ± 0.19 ^b^	4.19 ± 0.68 ^a^	0.72 ± 0.07 ^e^	3.14 ± 0.00 ^ab^	7.22 ± 0.22 ^b^	89.64 ± 0.22 ^c^	58.77 ± 0.28 ^c^
OS	14.34 ± 0.20 ^a^	1.92 ± 0.22 ^d^	0.92 ± 0.04 ^d^	3.58 ± 0.34 ^a^	8.12 ± 0.27 ^a^	88.30 ± 0.07 ^b^	62.92 ± 0.45 ^a^
HS	12.83 ± 0.56 ^b^	2.20 ± 0.39 ^c^	0.99 ± 0.07 ^c^	3.32 ± 0.27 ^ab^	7.63 ± 0.14 ^b^	89.05 ± 0.14 ^b^	60.62 ± 0.35 ^b^

Note: All data were expressed by mean ± SD (*n* = 2). Values with the different letters in the same column are significantly different (*p* < 0.05).

## Data Availability

The original contributions presented in the study are included in the article/Supplementary Materials, further inquiries can be directed to the corresponding authors.

## Supplementary Materials

The following supporting information can be downloaded at: https://www.mdpi.com/article/10.3390/foods14040706/s1, Table S1. Hydrolysis rates and Logarithm of Slope value of starch isolated from five different varieties of banana.
