# Supplementary material for: Physicochemical, Structural, and Digestive Properties of Green Banana Starch from Five Chinese Mutant Banana Species
Source: Foods. 2025 Feb 19;14(4):706. doi: 10.3390/foods14040706 (PMC11854689; doi:10.3390/foods14040706)
Supplement: Supplementary file 1 [file foods-14-00706-s001.zip › foods-3378620-supplementary.pdf]

Table S1. Hydrolysis rates and Logarithm of Slope value of starch isolated from five different varieties of banana

Time	Hydrolysis rates					ln(dC$_t$/d$_t$)				
s/min	NS	DS	GS	OS	HS	NS	DS	GS	OS	HS
0	0.06±0.00	0.12±0.04	0.12±0.00	0.22±0.26	0.22±0.26	-1.6889	-2.4521	-1.6281	-1.5963	-1.7556
5	0.98±0.16	0.55±0.13	1.10±0.00	1.23±0.12	1.08±0.09	-1.8048	-2.4507	-1.7045	-1.7246	-1.7736
10	1.70±0.12	0.99±0.05	1.93±0.04	2.00±0.19	1.91±0.04	-2.1102	-2.5245	-2.0409	-1.8527	-1.8492
15	2.19±0.00	1.35±0.03	2.40±0.12	2.80±0.10	2.65±0.04	-2.1843	-2.5249	-2.3510	-1.8734	-2.0197
20	2.83±0.00	1.88±0.19	2.89±0.00	3.53±0.07	3.24±0.00	-2.2103	-2.5435	-2.3510	-2.0010	-2.1836
30	3.75±0.24	2.50±0.19	3.81±0.16	4.76±0.03	4.32±0.04	-2.4691	-2.7301	-2.5036	-2.1828	-2.2997
45	4.91±0.08	3.39±0.15	4.88±0.12	6.30±0.30	5.71±0.17	-2.5276	-2.7873	-2.7121	-2.3402	-2.5358
60	6.15±0.20	4.40±0.29	5.80±0.20	7.65±0.13	6.70±0.00	-2.6838	-2.9301	-2.8031	-2.5730	-2.7605
90	7.76±0.13	5.64±0.30	7.59±0.20	9.53±0.18	8.52±0.13	-2.9033	-3.1682	-2.8518	-2.8672	-2.8452
120	9.44±0.04	6.87±0.14	9.27±0.20	11.07±0.19	10.18±0.13	-3.1510	-3.4483	-3.0241	-3.0441	-3.1299
150	10.33±0.09	7.54±0.03	10.51±0.24	12.39±0.24	11.14±0.26	-3.7684	-4.1086	-3.4052	-3.3281	-3.6216
180	10.82±0.12	7.85±0.14	11.26±0.16	13.22±0.20	11.79±0.39	-4.1135	-4.5766	-3.6881	-3.5878	-3.8351

Note: All data was expressed by mean ± SD (n = 2). Values with the different letters in the same column are significantly different ($p < 0.05$).
